# Unusual Revelation of an Anomalous Right Coronary Artery: Non-ST Segment Elevation Myocardial Infarction (NSTEMI) in the Context of Drug Abuse

**DOI:** 10.7759/cureus.91982

**Published:** 2025-09-10

**Authors:** Alec A Kadrie, Skyelor Black, Snehitha Balguri, Curtis Cary

**Affiliations:** 1 Internal Medicine, University of Tennessee Health Science Center College of Medicine, Memphis, USA; 2 Internal Medicine, University of Tennessee at Chattanooga, Chattanooga, USA

**Keywords:** anomalous right coronary artery, cocaine-induced vasospasm, coronary artery anomaly, interarterial course, non-st segment elevation myocardial infarction, polysubstance abuse, sudden cardiac death

## Abstract

Coronary artery anomalies (CAAs) are rare congenital abnormalities. Certain variants, such as those with an intramural course, are associated with increased risk of ischemia and sudden cardiac death, particularly under conditions of increased cardiac demand. We report the case of a 36-year-old male with polysubstance abuse who presented with chest pain after cocaine intake. Initial labs, testing, and imaging revealed respiratory failure, hypoxia, elevated cardiac biomarkers, and new-onset reduced ejection fraction. After the patient was medically stabilized, coronary computed tomography angiography was performed, which revealed an anomalous right coronary artery (RCA) originating from the left coronary cusp with a malignant intramural course and significant stenosis. The patient was then discharged and later underwent successful surgical marsupialization of the RCA into the aorta with cardiothoracic surgery. His postoperative recovery was uncomplicated, and he was discharged with close follow-up. In this case, it is likely that cocaine-induced vasospasm exacerbated the anomaly, leading to the non-ST segment elevation myocardial infarction. This case demonstrates the importance of considering coronary anomalies in young patients with chest pain and no traditional risk factors, particularly in the setting of stimulant use. Early detection and surgical intervention are critical to prevent sudden cardiac death, and multidisciplinary care is essential for long-term success.

## Introduction

Coronary artery anomalies (CAAs) represent a diverse group of rare congenital abnormalities involving the origin, course, or structure of the coronary arteries, with an estimated prevalence of 0.3% to 1% in the general population [1]. Among these, the anomalous aortic origin of the right coronary artery (RCA) is particularly rare, occurring in 0.026% to 0.25% of cases [1,2]. Variations of the RCA include ectopic origins from the pulmonary artery, aortic sinus of Valsalva, and left coronary cusp as well as intramural courses [3]. While many CAAs are benign, certain variations, such as anomalous aortic origins, carry a significant risk, including myocardial ischemia, sudden cardiac death (SCD), or other adverse events, particularly under conditions of increased cardiac demand [2,4,5]. Here, we present a rare case of an anomalous origin of the RCA from the left coronary cusp, which presented as a non-ST segment elevation myocardial infarction (NSTEMI) in the setting of polysubstance drug abuse.

## Case presentation

A 36-year-old male with no prior medical history other than polysubstance abuse presented to the emergency department after being found unresponsive in his car. He reported constant, sharp left-sided chest pain radiating to his neck, which was exacerbated by palpation and movement. He admitted to recent use of cocaine from a new source and an unknown pill. He denied any cardiac family history. Initial vital signs included a blood pressure of 99/58 mmHg, heart rate of 105 bpm, and oxygen saturation of 85% on 6L nasal cannula after Narcan administration. On examination, he was drowsy but arousable, with chest tenderness but no acute findings.

**Figure 1:**
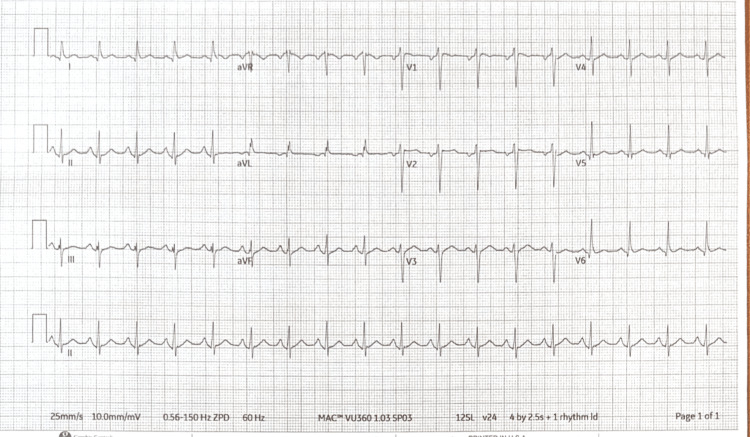
EKG demonstrating sinus tachycardia with the absence of any ischemic changes

Initial labs indicated hypercapnic respiratory failure, lactic acidosis, and a urine drug screen positive for benzodiazepines, cocaine, and fentanyl. High-sensitivity troponin levels were initially 122 ng/L, peaking at 948 ng/L, with a creatine phosphokinase level of 772 U/L (Table 1). ECG showed no ischemic changes (Figure 1), and transthoracic echocardiogram revealed new-onset reduced ejection fraction (35-40%) (Video 1).

**Table 1:** Laboratory findings with normal reference ranges

Laboratory Test	Patient Value	Reference Range
High-sensitivity troponin (initial)	122 ng/L	<14 ng/L
High-sensitivity troponin (peak)	948 ng/L	<14 ng/L
Creatine phosphokinase	772 U/L	26–192 U/L (may vary slightly by lab)
Arterial blood gas (PaCO₂)	53 mmHg	35–45 mmHg
Lactic acid	7.46 mmol/L	0.5–2.2 mmol/L
Urine drug screen	Positive for benzodiazepines, cocaine, fentanyl	Negative

**Video 1:** Transthoracic echocardiogram revealed new-onset reduced ejection fraction (35-40%)

**Figure 2:**
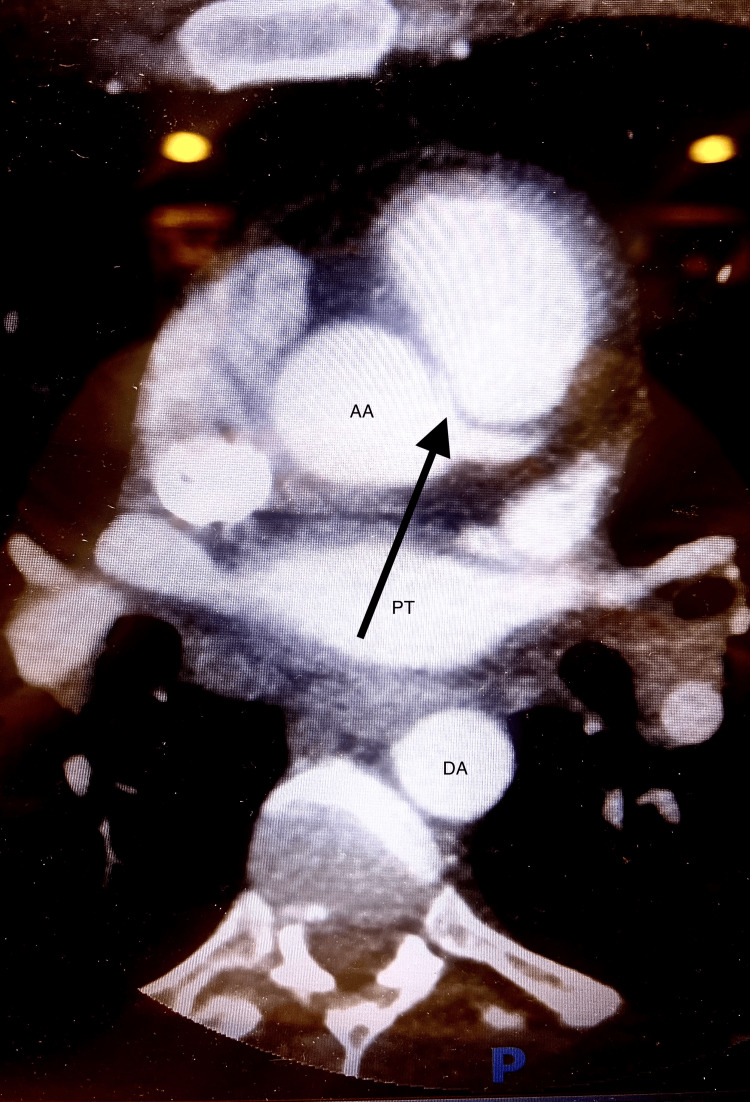
Coronary computed tomography angiography revealing an anomalous origin of the RCA originating from the left coronary cusp The arrow points to the anomalous origin of the RCA. AA, ascending aorta; DA, descending aorta; PT, pulmonary trunk; RCA, right coronary artery

The patient improved rapidly with fluid resuscitation and oxygen therapy (Vapotherm 40L at 45%, weaned to 2L nasal cannula). He was started on a heparin drip, aspirin, and metoprolol before undergoing a coronary computed tomography (CT) angiography. The scan revealed findings consistent with nonischemic cardiomyopathy as well as an anomalous origin of the RCA originating from the left coronary cusp, which then coursed anteriorly angiographically, with a moderate to severely stenotic interarterial course (Figure 2).

The patient stabilized and was discharged on carvedilol, losartan, and dapagliflozin with close follow-up. He was referred to outpatient cardiothoracic surgery, where he underwent RCA ostium repair with marsupialization into the aorta. The postoperative course was uneventful, and he was discharged on aspirin, clopidogrel, and rosuvastatin, with close cardiology and cardiothoracic surgery follow-up.

## Discussion

Ultimately, this case highlights a rare coronary anomaly, with a unique challenge to diagnosis and management. In our case, coronary CT angiography was performed in the setting of NSTEMI with polysubstance drug abuse and acute hypercapnic respiratory failure, revealing an anomalous origin of the RCA from the left coronary cusp. The relationship between the NSTEMI, CAA, and cocaine use is significant as cocaine-induced coronary vasospasm and the stenotic interarterial course of the RCA both likely contributed to the ischemic event.

Anomalous coronary arteries, though rare, should always be considered in young patients presenting with chest pain, particularly when traditional risk factors for ischemia are absent. Cocaine use, as in this case, can unmask or exacerbate underlying vulnerable coronary anomalies, complicating diagnosis and management. The stenotic interarterial course between the pulmonary artery and ascending aorta seen in our patient further increases the risk of compression, leading to exercise-induced angina, malignant arrhythmias, or SCD [2,6]. Recent fluid dynamics studies have shown that intramural or interarterial courses create abnormal hemodynamic conditions, including elevated wall shear stress and static pressure, particularly during exertion [7]. These pathophysiologic factors can predispose to vasospasm, endothelial dysfunction, and thrombosis, mechanisms that likely acted alongside cocaine-induced coronary vasospasm in our patient.

The American Heart Association (AHA)/American College of Cardiology (ACC) guidelines recommend surgical correction for anomalous aortic origins in patients with angina symptoms who present with evidence of myocardial ischemia in the matching territory or high-risk stenotic anatomy [3,8,9]. Additionally, surgery should be considered in asymptomatic patients with anomalous coronary arteries and evidence of myocardial ischemia or with high-risk anatomy [8-10].

Recent literature reports that post-surgical outcomes for these patients are favorable. In a cohort of 230 patients undergoing repair of anomalous coronary origins, there were no perioperative or late deaths, and only a 2.6% reoperation rate over a median four-year follow-up [11]. Another single-center study similarly found zero perioperative mortalities among 28 patients with interarterial courses [12]. While complication rates have been reported between 7% and 13%, Jegatheeswaran et al. suggest that surgical intervention substantially mitigates the risk of SCD and future ischemic events [13]. While not a strict requirement for surgical correction, achieving substance abstinence is essential for optimizing long-term cardiovascular health and surgical outcomes.

Our case also demonstrates the importance of a multidisciplinary approach in managing patients with CAAs and polysubstance use. Collaboration between cardiology, cardiothoracic surgery, medicine, and addiction specialists is critical to addressing both the immediate cardiac risks and the broader context of substance use [14]. Postoperative care and regular monitoring are integral to recovery, particularly in patients with a history of drug abuse, to prevent future complications and ensure adherence to lifestyle modifications and medical therapy.

By identifying and addressing both the coronary anomaly and the contributing role of polysubstance use, this case highlights the need for individualized and comprehensive care for patients presenting with rare cardiac conditions. Enhanced diagnostic algorithms may improve outcomes by identifying patients at risk and offering life-saving corrections.

## Conclusions

This case illustrates the connection between congenital coronary anomalies and substance abuse in the development of acute coronary syndromes. The anomalous origin of the RCA from the left coronary cusp with an interarterial course posed a high risk for ischemia, which was likely exacerbated by cocaine-induced vasospasm. Early recognition, multidisciplinary management, and timely surgical correction were key to the patient’s favorable outcome. This case emphasizes the importance of considering coronary anomalies in young patients presenting with chest pain, especially in the context of polysubstance use, and highlights the need for a tailored approach to diagnosis, treatment, and long-term follow-up.
